# The Woman Question

**Published:** 1873-04

**Authors:** D. L. D. Sheldon

**Affiliations:** New York


### THE WOMAN QUESTION.

### A REPLY.

Though women may generally admit the propriety of Mr. Ben. H. Pratt's views in regard to women, they will practically adopt the opposite view; the obvious reason being, that those who are termed the elite of society, ignore the dignity of labor, and discard the society of the poor; wealth without labor being the great elevator to supremacy, therefore to obtain desirable positions, either socially, politically or religiously, the proscribed have to play a deceitful game, by assuming, or as far as possible adopting the attributes and attire of wealth. Thus keeping themselves poor by assuming to be rich, and many prostitute themselves and children to secure the requisites for shielding their assumed position. Parents often adopt all manner of deceitful manœuvers to allure reputed wealth into nuptial alliance with their daughters, being either blind or unscrupulous in regard to this matrimonial prostitution—for it is nothing less, and until parents look upon this infamous sale instigated by themselves, in its proper light, the much needed reform, whereby honest labor may supercede wealth as a requisite to matrimonial ties, can never be perfected; because of such nuptial alliance being the main-spring of matrimonial infidelity and divorce. From the present erroneous idea in regard to labor, parents who well understand that the great object of life is connubial bliss, resulting in maternal responsibility, commencing with fœtal existance, attempt to shuffle off the responsibility of properly educating their daughters to fill this almost supreme position. How absurd to ignore the dignity of labor, by letting children grow up ignorant of the physical and mental benefits derived from and by labor, through which flow nearly all the necessaries and pleasures of life. Though woman's physical strength is comparative meekness, her power to influence man is almost unbounded, for it commences with fœtal existence, ending in death, unless eternal; thus demonstrating that the power behind the throne may be superior to the throne. Under these considerations, where is the advantage of female suffrage, which but few women desire? Cannot women as easily induce men to vote as they desire, as to induce them to grant them the power of doing the same thing? If so, why not save the trouble and expense of duplicating votes? Again, the real feminine woman generally abhors the idea of being brought in contact with the rough practices of corrupt politicians; it would not only harden her feelings of sympathy, but would diminish her influence, for woman's feminine characteristics constitute her loveliness, and therefore her ruling force; when this is properly applied, it is far superior to physical strength. Men of the highest talents and attainments, both physical and intellectual, not only succumb to this kind of influence, but they solicit it.—Married women, where real matrimonial affection exists, where love is compensated by love, do not require the suffrage power; they deem it inexpedient and unnecessary to take these new duties upon themselves at the expense of trusting their domestic affairs, and the rearing of children, in the charge of (may be,) unscrupulous servants, destitute of one pulsation of motherly love. Again, being disconnected with public politics, they avoid the apparent necessary excitement of politicians, therefore are much more capable of tendering wise counsel. Although there are as absolute differentiations of form, texture and characteristics between men and women as between the human being and the lower animals, and the positions to be held by them as absolute, in the aggregate, there is no superiority between the sexes, each equally depending upon the other for all the pleasures and advantages of life, and each possessing more of the qualities endearing to the other.

D. L. D. Sheldon, M. D.

*New York, March 4, 1873.*

